# Progress at Guy's Hospital

**Published:** 1903-03-28

**Authors:** 


### PROGRESS AT GUY'S HOSPITAL.

Guy's Hospital, thanks to the generous award of £5,000 annual subscription from King Edward's Hospital Fund, is now entirely open (with the exception of the pay wards) to the sick poor; and over 100 additional beds have been made available, bringing up the total to something more than 600. Alterations have been in progress for some time past, and the approaching summer months should see the completion of some of the more important details. Three new operating theatres are being provided on the floor between Martha and Lazarus Wards; Cornelius Ward is to be enlarged, and a Drunk or Dying Ward will be opened on the site of the present library. A magnificent library is being fitted up in light oak in what was formerly the nurses' home: this is entirely free of cost to the hospital, being the gift of one of the governors, Mr. Wills. It is to be called "The Wills Library." Job Ward, the reconstruction of which has been completed owing to the generous gift of £1,000 from Mrs. Walter Farquhar in memory of her late husband, has just been reopened for male surgical cases; and by rearrangement of the medical wards fifty additional beds have been made available—20 for men and 30 for women. The latter were badly needed.

